# Regional Differences in Individualism in Japan: Scoring Based on Family Structure

**DOI:** 10.3389/fpsyg.2020.01677

**Published:** 2020-07-21

**Authors:** Yuji Ogihara

**Affiliations:** ^1^Graduate School of Education, Kyoto University, Kyoto, Japan; ^2^Faculty of Science Division II, Tokyo University of Science, Tokyo, Japan

**Keywords:** individualism, regional difference, family structure, cultural change, regional variation, area difference, collectivism, culture

## Abstract

The present article reported regional (prefecture-level) differences in individualism in Japan based on family structure in 2005, 2010 and 2015. Previous research calculated 2005 prefecture-level scores of individualism-collectivism in Japan by analyzing five validated indicators of individualism-collectivism (divorce rate, percentage of people living alone, percentage of elderly people aged over 65 living alone, percentage of nuclear family households, and percentage of three-generation households). However, only the scores for 2005 had been presented. The scores and their regional differences may have changed over time. Therefore, the current article calculated individualism scores for 2010 and 2015 following previous research. Analyses showed that the scores were stable over time, indicating that regional differences in individualism were maintained for this period. This report is useful for understanding regional differences in psychological phenomena and validating new indicators at the regional level.

## Introduction

Nation is frequently used as the unit of analysis to examine the relationship between culture and psychology (e.g., US-Japan comparisons, cross-cultural research across 15 nations). Although using the nation as a unit of analysis is a common and effective method of research, sometimes an important fact may be ignored: variation exists not only between nations, but also within nations. Focusing on variation within a nation can provide insights for important questions such as what culture is, how culture and people make each other up, and how culture changes over time. People intuitively understand that nations have regional differences, but empirical evidence elucidating such differences is largely absent. Previous literature empirically has shown regional differences in some important psychological concepts within nation. For example, Vandello and Cohen (1999) provided evidence that individualism-collectivism scores differ between U.S. states.

### Regional Differences in Individualism in Japan

Intranational regional differences should be empirically investigated not only in the U.S., but also in other nations. Following previous research in the U.S. (Vandello and Cohen, 1999), Yamawaki (2012) reported that there is regional variation in individualism-collectivism within Japan by focusing on family structure. She used five indicators to calculate regional level individualism scores in Japan: divorce rate, the percentage of people living alone, the percentage of elderly people (over 65 years old) living alone, the percentage of nuclear family households, and the percentage of three-generation households. All of the indicators have been confirmed as valid indices of individualism-collectivism (e.g., Yamawaki, 2012; Ogihara, 2018b).

These scores are helpful for understanding regional variations in psychological phenomena. For example, experimental results that differ by region within a nation may reflect regional differences in individualism-collectivism (e.g., Kitayama et al., 2006). Further, capturing and calculating regional differences in individualism is useful for checking the validity of new indicators. Validity can be investigated not only at the individual or national levels, but also at the regional level. For example, when researchers have prefectural level data that is conceptually considered to reflect individualism, its validity can be empirically investigated by checking the relationship between the new data and the data of this report.

However, regional-level individualism scores for Japan have only been reported for data collected in 2005 (Yamawaki, 2012). Thus, the scores after 2005 are unclear. Because family structure can change over time, individualism scores and their regional-level differences can also change over time. Indeed, Ogihara (2018b) analyzed temporal changes in the same indicators at the national level in Japan between 1947 and 2015, and found that the divorce rate, the rate of people living alone (within both the total population and the elderly population aged over 65), and the rate of nuclear households increased, while the rate of three-generation households and the household size decreased. These results indicated that people came to live more independently from other family members and that family structure became more individual-based, suggesting an increase in individualism in Japan (also see Ogihara et al., 2015; for reviews, see Ogihara, 2017, 2018a). These changes may occur to a greater extent in certain prefectures than others. Therefore, it is necessary to update the individualism scores and examine how they vary by region. Because the Japanese population census is conducted every 5 years, the scores for 2010 and 2015 were calculated in this research.

### The Current Report

I report regional-level individualism scores in Japan focusing on family structure and using the same methodology as the previous work (Yamawaki, 2012). Japan is divided into 47 prefectures, which are subnational jurisdictions geographically bigger than cities and towns and analogous to states in the United States.

## Method

### Indicators

I used the five indicators used in previous research (Yamawaki, 2012): divorce rate, percentage of people living alone, percentage of elderly people aged over 65 living alone^1^, percentage of nuclear family households, and percentage of three-generation households (reverse-scored).

The divorce rate data came from the Ministry of Health, Labour and Welfare (2019) and the data for the other four indicators came from the Statistics Bureau of Japan (2019). All data used in this research are available online (https://doi.org/10.17605/OSF.IO/MQBRU).

#### The Divorce-to-Marriage Ratio and the Divorce-to-Population Ratio

Whereas previous research used the divorce-to-marriage ratio as the divorce rate indicator (Yamawaki, 2012), the current research also used the divorce-to-population ratio (rate of divorce per 1,000 people, i.e., the crude divorce rate).

#### 2005

The divorce-to-population ratio was moderately associated with the other four indicators (Table 1). The divorce-to-marriage ratio was moderately to weakly related to the other four indicators^2^. The reliability score was higher (Cronbach's α = 0.89; McDonald's ω = 0.90) when using the divorce-to-population ratio than when using the divorce-to-marriage ratio (α = 0.82; ω = 0.86).

**Table 1:** Correlation coefficients among indicators.

	**1. Divorce rate (marriage)**	**2. Divorce rate (population)**	**3. Percentage of people living alone**	**4. Percentage of elderly people aged over 65 living alone**	**5. Percentage of nuclear family households**	**6. Percentage of** **three-generation households (R)**
**(A) 2005**						
1. Divorce rate (marriage)	–	0.62	0.14	0.42	0.21	0.25
2. Divorce rate (population)		–	0.51	0.60	0.49	0.68
3. Percentage of people living alone			–	0.84	0.16	0.79
4. Percentage of elderly people aged over 65 living alone				–	0.46	0.87
5. Percentage of nuclear family households					–	0.72
6. Percentage of three-generation households (R)						–
**(B) 2010**						
1. Divorce rate (marriage)	–	0.48	−0.05	0.22	0.19	0.10
2. Divorce rate (population)		–	0.51	0.58	0.43	0.68
3. Percentage of people living alone			–	0.87	0.03	0.81
4. Percentage of elderly people aged over 65 living alone				–	0.32	0.87
5. Percentage of nuclear family households					–	0.61
6. Percentage of three-generation households (R)						–
**(C) 2015**						
1. Divorce rate (marriage)	–	0.51	0.00	0.33	0.38	0.25
2. Divorce rate (population)		–	0.49	0.66	0.39	0.71
3. Percentage of people living alone			–	0.89	−0.15	0.79
4. Percentage of elderly people aged over 65 living alone				–	0.14	0.87
5. Percentage of nuclear family households					–	0.47
6. Percentage of three-generation households (R)						–

*The divorce rate (marriage) indicates divorce-to-marriage ratio while the divorce rate (population) indicates divorce-to-population ratio. “(R)” indicates a reversed score. N = 47*.

#### 2010

The correlation pattern found in 2010 was similar to the pattern in 2005. The divorce-to-population ratio was moderately associated with the other four indicators (Table 1). The divorce-to-marriage ratio was weakly correlated with the three indicators (the percentage of elderly people aged over 65 living alone, the rate of nuclear households, and the rate of three-generation households), but was not correlated with the percentage of people living alone. The reliability score for the divorce-to-population ratio (α = 0.87; ω = 0.89) was higher than that for the divorce-to-marriage ratio (α = 0.77; ω = 0.82).

#### 2015

The correlation pattern found in 2015 was also similar to the patterns in 2005 and 2010. The divorce-to-population was moderately to highly correlated with the other four indicators (Table 1). The divorce-to-marriage ratio was moderately to weakly related to the three indicators (the percentage of elderly people aged over 65 living alone, the rate of nuclear households, and the rate of three-generation households), but was not related to the percentage of people living alone. The reliability score was higher for the divorce-to-population ratio (α = 0.85; ω = 0.88) than for the divorce-to-marriage ratio (α = 0.77; ω = 0.82).

#### Relationships Among Indicators

Correlations among prefecture-level indicators in Japan at the three time points (2005, 2010, and 2015) are shown in Table 1. Overall, the four indicators except for the divorce rate were highly to moderately correlated with each other, suggesting that the indicators measure a consistent concept. The correlations between the rate of people living alone and the rate of nuclear households were small or close to zero^3^. In 2015, the correlation between the rate of people aged over 65 living alone and the rate of nuclear households was small.

### Scoring

Aggregating five indicators into one index is helpful because each indicator has its own random errors but this aggregation can decrease random errors, thereby creating a more accurate measure of individualism. In this report, I computed two types of aggregated scores: averaged z-scores and Principal Component Scores (PCSs).

#### Averaged Z-Scores

Following previous research (Yamawaki, 2012), the averaged z-scores for each of the 47 prefectures were calculated by averaging^4^ the z-scores of the five indicators^5^.

#### Principal Component Scores (PCSs)

In the original study (Yamawaki, 2012), the indicators were simply averaged, where each of the five indicators were considered to contribute equally to the overall individualism-collectivism score. However, the associations between each of the five indicators and the overall individualism score may differ (e.g., the divorce rate might reflect individualism more than the percentage of nuclear family households). Thus, I conducted a principal component analysis (PCA) and used the principal component scores (PCSs) as an aggregate indicator when the divorce-to-population ratio was used as an indicator of divorce rate^6^. Aggregate z-scores and aggregate PCSs were highly correlated (Table 2).

**Table 2:** Correlation coefficients among aggregated scores (z-scores and principal component scores).

**(A) DIVORCE-TO-MARRIAGE RATIO**						
	**z-score 2005**	**z-score 2010**	**z-score 2015**			
z-score 2005	–	0.990	0.978			
z-score 2010	0.980	–	0.990			
z-score 2015	0.970	0.989	–			
**(B) DIVORCE-TO-POPULATION RATIO**						
	**z-score 2005**	**z-score 2010**	**z-score 2015**	**PCS 2005**	**PCS 2010**	**PCS 2015**
z-score 2005	–	0.994	0.990	0.999	0.995	0.993
z-score 2010	0.994	–	0.996	0.992	0.997	0.990
z-score 2015	0.989	0.990	–	0.987	0.990	0.989
PCS 2005	0.999	0.992	0.988	–	0.996	0.994
PCS 2010	0.995	0.996	0.988	0.997	–	0.996
PCS 2015	0.993	0.989	0.991	0.995	0.995	–

*PCS, Principal Component Score. Scores in the upper half represent Pearson's correlation coefficients, while those in the lower half represent Spearman's correlation coefficients. Both results were consistent*.

### Score Stability

To examine the longitudinal stability of the overall scores, the correlations between the scores were calculated at the three time points, for both the averaged z-scores and PCSs (Table 2). Both the Pearson's and Spearman's correlations were very high, suggesting that the scores were stable and their ranks were almost fixed across the years (also see, the prefectural ranking of individualism at each time point in Table S1 in the Supplementary Material).

## Conclusion

The present article reported regional (prefecture-level) differences in individualism in Japan based on family structure^7^. Following previous research (Yamawaki, 2012), the aggregated scores of five indicators in 2005, 2010, and 2015 were calculated. The two types of aggregated scores were stable over time, indicating that the prefecture-level differences in individualism were stable. This report is helpful to understand prefecture-level differences in psychological phenomena in Japan. Moreover, the scores are useful for examining the validity of new indicators at the prefectural level.

## Data Availability Statement

All datasets generated for this study are included in the article/Supplementary Material.

## Author Contributions

The author confirms being the sole contributor of this work and has approved it for publication.

## Conflict of Interest

The author declares that the research was conducted in the absence of any commercial or financial relationships that could be construed as a potential conflict of interest.

## Footnotes

^1^Percentage of elderly people aged over 65 living alone conceptually and numerically overlaps with percentage of people living alone. This overlap was also found in prior research (Vandello and Cohen, 1999; Yamawaki, 2012). Thus, I checked whether the results were unchanged even when the percentage of people aged over 65 living alone was excluded from the analyses. I found that the patterns were consistent.

^2^These small correlations were also reported in prior research (Vandello and Cohen, 1999; Yamawaki, 2012).

^3^These small correlations were also found in past research (Vandello and Cohen, 1999; Yamawaki, 2012).

^4^The original paper (Yamawaki, 2012) described the aggregation calculation as follows: “[t]he overall collectivism score was the sum of the individual z scores for each of the five items. The z scores were then transformed by multiplying the means by 20 and adding this result to 50, thereby resulting in a collectivism score that would be positive and range from approximately 1 to 100” (p. 1194). However, if the overall scores were indeed based on the “sum” of the individual z-scores, the range of the transformed scores should be much larger. Thus, the scores seemed to be based on the “average” of the individual z-scores for each of the five indicators.

^5^To ensure that the data and calculations were exactly same as in the original study (Yamawaki, 2012), I attempted to directly replicate the original study. Although the overall scores were very similar, they were not identical (the Pearson's correlation coefficient between the scores in the original study and the present report was 0.89 and Spearman's rank-order correlation coefficient was 0.88). Prior to the current study, another independent researcher in Japan familiar with this topic also tried to replicate her study, but also failed (similar, but not identical scores were obtained). Thus, I asked Dr. Yamawaki to directly replicate her results by re-calculating her scores and to discuss why my replication attempt was unsuccessful. However, she declined my request (the prior independent researcher also made a similar request, but was similarly unsuccessful). Although the other researcher and I have tried to understand why our calculations differ from those in the original study, we have not been able to reach an answer. Of course, it is desirable to discover the reason, but considering that we had no way and the scores were very similar, we used the scores we calculated in this report.

^6^I did not conduct PCA when the divorce-to-marriage ratio was used as an indicator of divorce rate because the correlations between the divorce-to-marriage ratio and the other four indicators were not sufficiently large.

^7^The dataset focused on family structure. Although family structure is an important aspect of individualism-collectivism, it is not the only one (e.g., Ogihara, 2017). Thus, it is desirable to examine regional differences in individualism-collectivism using other indicators in future research.
