# Supplementary material for: Regional Differences in Individualism in Japan: Scoring Based on Family Structure
Source: Front Psychol. 2020 Jul 21;11:1677. doi: 10.3389/fpsyg.2020.01677 (PMC7385408; doi:10.3389/fpsyg.2020.01677)
Supplement: Supplementary file 1 [file Table_1.DOCX]

**Supplementary Material**

*Table S1.* Prefectural ranking of individualism in Japan.

(A) Averaged z-scores (divorce-to-marriage ratio)

|  | 2005 | | 2010 | | 2015 | |
| --- | --- | --- | --- | --- | --- | --- |
| Rank | Prefecture | Score | Prefecture | Score | Prefecture | Score |
| 1 | Kagoshima | 1.402 | Hokkaido | 1.246 | Kagoshima | 1.227 |
| 2 | Hokkaido | 1.234 | Kagoshima | 1.239 | Hokkaido | 1.193 |
| 3 | Kochi | 1.232 | Osaka | 1.191 | Kochi | 1.107 |
| 4 | Osaka | 1.215 | Kochi | 1.036 | Miyazaki | 1.089 |
| 5 | Tokyo | 1.002 | Miyazaki | 0.829 | Osaka | 1.030 |
| 6 | Miyazaki | 0.949 | Wakayama | 0.772 | Wakayama | 0.856 |
| 7 | Ehime | 0.783 | Ehime | 0.762 | Yamaguchi | 0.791 |
| 8 | Okinawa | 0.714 | Tokyo | 0.716 | Okinawa | 0.688 |
| 9 | Fukuoka | 0.703 | Okinawa | 0.644 | Ehime | 0.654 |
| 10 | Hyogo | 0.659 | Hyogo | 0.615 | Hyogo | 0.597 |
| 11 | Wakayama | 0.658 | Fukuoka | 0.567 | Tokyo | 0.558 |
| 12 | Yamaguchi | 0.658 | Yamaguchi | 0.558 | Kanagawa | 0.520 |
| 13 | Kyoto | 0.604 | Kyoto | 0.554 | Fukuoka | 0.504 |
| 14 | Nagasaki | 0.546 | Kanagawa | 0.523 | Hiroshima | 0.478 |
| 15 | Kanagawa | 0.539 | Hiroshima | 0.493 | Kyoto | 0.468 |
| 16 | Hiroshima | 0.534 | Saitama | 0.383 | Nara | 0.460 |
| 17 | Oita | 0.393 | Nara | 0.363 | Oita | 0.390 |
| 18 | Saitama | 0.301 | Oita | 0.350 | Saitama | 0.358 |
| 19 | Chiba | 0.262 | Nagasaki | 0.347 | Chiba | 0.329 |
| 20 | Tokushima | 0.160 | Chiba | 0.283 | Nagasaki | 0.288 |
| 21 | Nara | 0.149 | Kagawa | 0.198 | Kagawa | 0.222 |
| 22 | Kagawa | 0.132 | Gunma | 0.095 | Gunma | 0.095 |
| 23 | Aomori | 0.105 | Tokushima | 0.092 | Kumamoto | 0.092 |
| 24 | Okayama | 0.018 | Yamanashi | 0.059 | Yamanashi | 0.004 |
| 25 | Kumamoto | -0.009 | Kumamoto | 0.025 | Mie | -0.002 |
| 26 | Mie | -0.027 | Okayama | -0.002 | Tokushima | -0.006 |
| 27 | Aichi | -0.052 | Aomori | -0.023 | Okayama | -0.087 |
| 28 | Yamanashi | -0.113 | Mie | -0.046 | Aichi | -0.125 |
| 29 | Gunma | -0.147 | Aichi | -0.091 | Aomori | -0.216 |
| 30 | Miyagi | -0.440 | Miyagi | -0.351 | Ibaraki | -0.297 |
| 31 | Ibaraki | -0.456 | Ibaraki | -0.357 | Shizuoka | -0.391 |
| 32 | Saga | -0.456 | Tochigi | -0.453 | Shiga | -0.437 |
| 33 | Shizuoka | -0.502 | Shizuoka | -0.481 | Tochigi | -0.444 |
| 34 | Tochigi | -0.571 | Nagano | -0.533 | Miyagi | -0.528 |
| 35 | Ishikawa | -0.620 | Shiga | -0.573 | Nagano | -0.531 |
| 36 | Shiga | -0.634 | Tottori | -0.590 | Ishikawa | -0.534 |
| 37 | Nagano | -0.636 | Fukushima | -0.618 | Akita | -0.590 |
| 38 | Tottori | -0.639 | Ishikawa | -0.652 | Gifu | -0.642 |
| 39 | Fukushima | -0.685 | Iwate | -0.658 | Tottori | -0.674 |
| 40 | Iwate | -0.755 | Akita | -0.668 | Saga | -0.678 |
| 41 | Gifu | -0.802 | Saga | -0.700 | Fukushima | -0.785 |
| 42 | Shimane | -0.876 | Gifu | -0.768 | Iwate | -0.789 |
| 43 | Akita | -0.952 | Shimane | -0.891 | Shimane | -0.836 |
| 44 | Toyama | -1.194 | Toyama | -1.186 | Toyama | -1.127 |
| 45 | Niigata | -1.319 | Fukui | -1.312 | Niigata | -1.190 |
| 46 | Fukui | -1.335 | Niigata | -1.319 | Fukui | -1.289 |
| 47 | Yamagata | -1.730 | Yamagata | -1.667 | Yamagata | -1.802 |

(B-1) Principal component scores (divorce-to-population ratio)

|  | 2005 | | 2010 | | 2015 | |
| --- | --- | --- | --- | --- | --- | --- |
| Rank | Prefecture | Score | Prefecture | Score | Prefecture | Score |
| 1 | Tokyo | 1.899 | Tokyo | 1.953 | Tokyo | 2.052 |
| 2 | Osaka | 1.771 | Osaka | 1.868 | Osaka | 1.716 |
| 3 | Kagoshima | 1.620 | Hokkaido | 1.554 | Hokkaido | 1.542 |
| 4 | Hokkaido | 1.494 | Kagoshima | 1.548 | Kagoshima | 1.532 |
| 5 | Okinawa | 1.311 | Okinawa | 1.258 | Okinawa | 1.446 |
| 6 | Kochi | 1.230 | Fukuoka | 1.084 | Kochi | 1.199 |
| 7 | Miyazaki | 1.099 | Kanagawa | 1.038 | Fukuoka | 1.133 |
| 8 | Fukuoka | 1.059 | Miyazaki | 0.986 | Miyazaki | 1.108 |
| 9 | Kanagawa | 1.042 | Kochi | 0.961 | Kanagawa | 0.959 |
| 10 | Hyogo | 0.866 | Kyoto | 0.861 | Kyoto | 0.864 |
| 11 | Kyoto | 0.840 | Hyogo | 0.849 | Wakayama | 0.744 |
| 12 | Ehime | 0.827 | Ehime | 0.802 | Hyogo | 0.742 |
| 13 | Hiroshima | 0.765 | Hiroshima | 0.790 | Yamaguchi | 0.707 |
| 14 | Yamaguchi | 0.686 | Wakayama | 0.680 | Hiroshima | 0.703 |
| 15 | Wakayama | 0.556 | Yamaguchi | 0.604 | Ehime | 0.691 |
| 16 | Chiba | 0.511 | Chiba | 0.535 | Oita | 0.445 |
| 17 | Saitama | 0.487 | Saitama | 0.522 | Chiba | 0.433 |
| 18 | Nagasaki | 0.442 | Oita | 0.462 | Saitama | 0.314 |
| 19 | Oita | 0.427 | Aichi | 0.324 | Kagawa | 0.268 |
| 20 | Aichi | 0.242 | Nagasaki | 0.245 | Aichi | 0.251 |
| 21 | Kagawa | 0.134 | Kagawa | 0.182 | Nagasaki | 0.245 |
| 22 | Okayama | 0.027 | Nara | 0.078 | Kumamoto | 0.052 |
| 23 | Nara | -0.037 | Okayama | 0.028 | Okayama | 0.004 |
| 24 | Kumamoto | -0.061 | Kumamoto | -0.013 | Nara | -0.080 |
| 25 | Mie | -0.063 | Yamanashi | -0.062 | Mie | -0.087 |
| 26 | Tokushima | -0.111 | Gunma | -0.083 | Yamanashi | -0.101 |
| 27 | Yamanashi | -0.173 | Mie | -0.091 | Tokushima | -0.122 |
| 28 | Gunma | -0.198 | Tokushima | -0.117 | Gunma | -0.133 |
| 29 | Aomori | -0.234 | Miyagi | -0.365 | Miyagi | -0.354 |
| 30 | Miyagi | -0.433 | Aomori | -0.445 | Shizuoka | -0.494 |
| 31 | Shizuoka | -0.480 | Shizuoka | -0.462 | Aomori | -0.501 |
| 32 | Tochigi | -0.533 | Tochigi | -0.466 | Ibaraki | -0.510 |
| 33 | Ibaraki | -0.581 | Ibaraki | -0.515 | Tochigi | -0.541 |
| 34 | Shiga | -0.642 | Shiga | -0.558 | Shiga | -0.573 |
| 35 | Ishikawa | -0.711 | Ishikawa | -0.714 | Ishikawa | -0.631 |
| 36 | Nagano | -0.719 | Nagano | -0.772 | Nagano | -0.759 |
| 37 | Saga | -0.763 | Tottori | -0.817 | Tottori | -0.765 |
| 38 | Tottori | -0.849 | Saga | -0.928 | Fukushima | -0.830 |
| 39 | Fukushima | -0.895 | Fukushima | -0.937 | Saga | -1.015 |
| 40 | Gifu | -1.048 | Gifu | -1.051 | Gifu | -1.046 |
| 41 | Iwate | -1.097 | Iwate | -1.107 | Shimane | -1.062 |
| 42 | Shimane | -1.221 | Shimane | -1.151 | Iwate | -1.100 |
| 43 | Akita | -1.475 | Akita | -1.344 | Akita | -1.294 |
| 44 | Toyama | -1.537 | Toyama | -1.599 | Toyama | -1.546 |
| 45 | Fukui | -1.543 | Niigata | -1.670 | Fukui | -1.624 |
| 46 | Niigata | -1.702 | Fukui | -1.710 | Niigata | -1.645 |
| 47 | Yamagata | -2.227 | Yamagata | -2.238 | Yamagata | -2.339 |

(B-2) Averaged z-scores (divorce-to-population ratio)

|  | 2005 | | 2010 | | 2015 | |
| --- | --- | --- | --- | --- | --- | --- |
| Rank | Prefecture | Score | Prefecture | Score | Prefecture | Score |
| 1 | Osaka | 1.464 | Osaka | 1.472 | Osaka | 1.273 |
| 2 | Tokyo | 1.455 | Tokyo | 1.304 | Okinawa | 1.220 |
| 3 | Kagoshima | 1.316 | Kagoshima | 1.243 | Kagoshima | 1.211 |
| 4 | Hokkaido | 1.238 | Hokkaido | 1.239 | Tokyo | 1.189 |
| 5 | Okinawa | 1.147 | Okinawa | 1.111 | Hokkaido | 1.151 |
| 6 | Kochi | 0.984 | Kanagawa | 0.853 | Miyazaki | 0.960 |
| 7 | Miyazaki | 0.935 | Miyazaki | 0.847 | Kochi | 0.833 |
| 8 | Kanagawa | 0.877 | Fukuoka | 0.819 | Kanagawa | 0.789 |
| 9 | Fukuoka | 0.864 | Hyogo | 0.728 | Fukuoka | 0.786 |
| 10 | Hyogo | 0.741 | Kochi | 0.705 | Wakayama | 0.716 |
| 11 | Ehime | 0.678 | Ehime | 0.655 | Hyogo | 0.658 |
| 12 | Kyoto | 0.654 | Hiroshima | 0.628 | Yamaguchi | 0.576 |
| 13 | Hiroshima | 0.621 | Kyoto | 0.618 | Hiroshima | 0.564 |
| 14 | Yamaguchi | 0.553 | Wakayama | 0.609 | Kyoto | 0.550 |
| 15 | Wakayama | 0.492 | Saitama | 0.535 | Ehime | 0.539 |
| 16 | Saitama | 0.470 | Yamaguchi | 0.486 | Saitama | 0.430 |
| 17 | Chiba | 0.467 | Chiba | 0.486 | Chiba | 0.428 |
| 18 | Nagasaki | 0.368 | Oita | 0.358 | Oita | 0.336 |
| 19 | Oita | 0.335 | Aichi | 0.277 | Kagawa | 0.244 |
| 20 | Aichi | 0.206 | Nara | 0.225 | Nara | 0.233 |
| 21 | Kagawa | 0.126 | Nagasaki | 0.204 | Aichi | 0.221 |
| 22 | Nara | 0.037 | Kagawa | 0.170 | Nagasaki | 0.205 |
| 23 | Okayama | 0.008 | Okayama | 0.018 | Kumamoto | 0.049 |
| 24 | Mie | -0.022 | Gunma | 0.015 | Mie | 0.034 |
| 25 | Kumamoto | -0.057 | Yamanashi | -0.004 | Gunma | 0.028 |
| 26 | Tokushima | -0.109 | Kumamoto | -0.013 | Okayama | -0.006 |
| 27 | Gunma | -0.124 | Mie | -0.017 | Yamanashi | -0.008 |
| 28 | Yamanashi | -0.129 | Tokushima | -0.116 | Tokushima | -0.148 |
| 29 | Aomori | -0.183 | Shizuoka | -0.334 | Ibaraki | -0.308 |
| 30 | Shizuoka | -0.375 | Ibaraki | -0.345 | Shiga | -0.317 |
| 31 | Miyagi | -0.375 | Miyagi | -0.345 | Shizuoka | -0.320 |
| 32 | Tochigi | -0.417 | Tochigi | -0.354 | Tochigi | -0.367 |
| 33 | Ibaraki | -0.439 | Aomori | -0.384 | Miyagi | -0.436 |
| 34 | Shiga | -0.506 | Shiga | -0.400 | Aomori | -0.469 |
| 35 | Nagano | -0.589 | Nagano | -0.593 | Nagano | -0.526 |
| 36 | Saga | -0.613 | Ishikawa | -0.624 | Ishikawa | -0.527 |
| 37 | Ishikawa | -0.629 | Tottori | -0.697 | Tottori | -0.664 |
| 38 | Tottori | -0.724 | Saga | -0.725 | Gifu | -0.698 |
| 39 | Fukushima | -0.740 | Fukushima | -0.776 | Saga | -0.754 |
| 40 | Gifu | -0.845 | Gifu | -0.790 | Fukushima | -0.780 |
| 41 | Iwate | -0.939 | Iwate | -0.959 | Shimane | -0.958 |
| 42 | Shimane | -1.072 | Shimane | -1.012 | Iwate | -0.998 |
| 43 | Akita | -1.255 | Akita | -1.129 | Akita | -1.118 |
| 44 | Toyama | -1.285 | Toyama | -1.302 | Toyama | -1.195 |
| 45 | Fukui | -1.290 | Niigata | -1.393 | Fukui | -1.320 |
| 46 | Niigata | -1.439 | Fukui | -1.414 | Niigata | -1.342 |
| 47 | Yamagata | -1.881 | Yamagata | -1.880 | Yamagata | -1.964 |
